# Structure and concept of ICU rounds: the VIS-ITS survey

**DOI:** 10.1007/s00063-021-00830-3

**Published:** 2021-06-14

**Authors:** Bastian Hillmann, Daniel Schwarzkopf, Tanja Manser, Christian Waydhas, Reimer Riessen

**Affiliations:** 1 Department of Psychiatry and Psychotherapy, University of Tübingen, Tübingen, Germany; 2 Center for Sepsis Control and Care, University of Jena, Jena, Germany; 3 FHNW School of Applied Psychology, University of Applied Sciences and Arts Northwestern Switzerland, Olten, Switzerland; 4 Surgical ICU, Klinikum Bergmannsheil, University of Bochum, Bochum, Germany; 5 Medical Faculty, University of Duisburg-Essen, Essen, Germany; 6 Medical ICU, Department of Medicine, University of Tübingen, Otfried-Müller-Str. 10, 72076 Tübingen, Germany

**Keywords:** Daily goals, Decision-maker, Intensive care unit, Electronic health record, Patient data managment system, Intensivmediziner, Tagesziele, Patientenversorgung, Elektronische Kurve, Visitendauer

## Abstract

**Objective:**

To gather data about structural and procedural characteristics of patient rounds in the intensive care unit (ICU) setting.

**Design:**

A structured online survey was offered to members of two German intensive care medicine societies.

**Measurements and main results:**

Intensivists representing 390 German ICUs participated in this study (university hospitals 25%, tertiary hospitals 23%, secondary hospitals 36%, primary hospitals 16%). In 90% of participating ICUs, rounds were reported to take place in the morning and cover an average of 12 intensive care beds and 6 intermediate care beds within 60 min. With an estimated bed occupancy of 80%, this averaged to 4.3 min spent per patient during rounds. In 96% of ICUs, rounds were stated to include a bedside visit. On weekdays, 86% of the respondents reported holding a second ICU round with the attendance of a qualified decision-maker (e.g. board-certified intensivist). On weekends, 79% of the ICUs performed at least one round with a decision-maker per day. In 18%, only one ICU round per weekend was reported, mostly on Sundays. The highest-qualified decision-maker present during rounds on most ICUs was an ICU attending (57%). Residents (96%) and intensive care nurses (87%) were stated to be always or usually present during rounds. In contrast, physiotherapists, respiratory therapists or medical specialists such as pharmacists or microbiologist were not regular members of the rounding team on most ICUs. In the majority of cases, the participants reported examining the medical chart directly before or during the bedside visit (84%). An electronic patient data management system (PDMS) was available on 31% of ICUs. Daily goals were always (55%) or usually (39%) set during rounds.

**Conclusion:**

This survey gives a broad overview of the structure and processes of ICU rounds in different sized hospitals in Germany. Compared to other mostly Anglo-American studies, German ICU rounds appear to be shorter and less interdisciplinary.

## Abstract (de)

**Zielstellung:**

Informationen über Strukturen und Abläufe intensivmedizinischer Visiten in Deutschland zu sammeln.

**Methodik:**

Ein strukturierter Onlinefragebogen, versendet an die Mitglieder zweier großer intensivmedizinischer Fachgesellschaften in Deutschland.

**Ergebnisse:**

Mitarbeiter von 390 deutschen Intensivstationen nahmen an dieser Studie teil (Universitätskliniken 25 %, Maximalversorger 23 %, Regionalversorger 36 %, Grundversorger 16 %).

Die meisten (90 %) der teilnehmenden Intensivstationen visitierten am Morgen durchschnittlich 12 Intensivbetten und 6 Intermediate-Care-Betten in 60 min. Bei einer angenommenen Bettenauslastung von 80 % lässt sich eine Visitenzeit von 4,3 min pro Patient berechnen. 96 % der Stationen berichteten, dass die Visite den Besuch des Patienten am Krankenbett beinhaltete. Wochentags führten 86 % der Stationen täglich eine zweite Visite mit Anwesenheit eines qualifizierten Entscheidungsträgers (beispielsweise ein Facharzt mit Zusatzbezeichnung Intensivmedizin) durch. Am Wochenende führten 79 % der Stationen mindestens eine Visite pro Tag mit Anwesenheit eines Entscheidungsträgers durch. In 18 % der Fälle wurde nur eine Visite pro Wochenende, meistens sonntags, durchgeführt.

Der am häufigsten anwesende Entscheidungsträger bei intensivmedizinischen Visiten war der Oberarzt mit Intensivweiterbildung (57 %). Assistenzärzte (96 %) und Intensivpflegekräfte (87 %) wurden als immer oder meistens anwesend benannt. Im Gegensatz dazu waren auf den meisten Intensivstationen Physiotherapeuten, Atmungstherapeuten, Pharmazeuten oder Mikrobiologen keine regulären Mitglieder des Visitenteams. In den meisten Fällen gaben Teilnehmende an, die Patientenkurve direkt vor oder während der Visite am Krankenbett zu sichten (84 %). Ein elektronisches Patientendatenmanagementsystem (PDMS) war auf 31 % der Intensivstationen verfügbar. Tagesziele wurden immer (55 %) oder meistens (39 %) während der Visite festgelegt.

**Fazit:**

Die Ergebnisse dieses Fragebogens geben einen breiten Überblick über die Strukturen und Abläufe der intensivmedizinischen Visiten in Krankenhäusern unterschiedlicher Versorgungsstufen in Deutschland. Verglichen mit Daten aus angloamerikanischen Studien scheinen deutsche Intensivvisiten kürzer und weniger interdisziplinär zu sein.

## Introduction

Rounds are a central part of daily clinical routine on almost every ICU globally. They play a key role in communication within the ICU team and with patients and their families. During rounds, the aim should be to discuss all essential diagnostic, therapeutic and organizational aspects of patient care in a structured manner, including the documentation of daily goals [1]. Rounds have an important influence on patient safety and play a vital role in quality management on the ICU [2]. Rounding features are good starting points when planning to improve ICU performance [3].

However, there are hardly any standards or guidelines as to how ICU rounds should be structured and performed. In 2013 Lane et al. [4] published a first systematic review that provided 13 best practices for ICU rounds. In a 2015 published survey conducted using 111 Canadian ICUs, Holodinsky et al. [5] described considerable variations in rounding practices and several opportunities for improvement. No comparable data is available characterizing ICU rounds in other parts of the world.

In this survey, we asked intensivists from a wide spectrum of German ICUs to provide detailed information about their rounding practices and environment. The findings of this study could serve as a basis for an evaluation of individual rounding routines and as an initiative to improve ICU rounds.

## Materials and methods

### Study design

The study was designed as a cross-sectional survey and was reviewed by the ethics committee of the University of Tübingen, which waived the need for informed consent. The description of this study follows recommendations given in the CHERRIES checklist (Checklist for Reporting Results of Internet E‑Surveys) [6].

### Survey

A standardized survey was developed considering the few aforementioned studies that exist on this topic [4, 5]. Besides those, no other guidelines or references on ICU rounding could be found. The survey was implemented using an online-based survey tool (“Unipark”) [7]. The survey was structured into five main topics: demographic information of the participant, structural characteristics of the ICU, rounding structure and processes, rounds of external treatment teams and handovers. In order to allow participants to describe the various aspects of their rounding processes, different types of questions were implemented in the survey: single choice, multiple choice, 5‑point scale and open answer questions. Participants also had the opportunity to add information about the rounding structure or process on every page, if the predefined options did not reflect it.

All questions and answering options were reviewed and optimized in an iterative procedure by all authors, and finally, pilot-tested by 5 intensivists.

### Procedure

The survey was primarily addressed to ICU executives of all intensive care specialties. The survey was distributed via the e‑mail networks of two major German intensive care medicine societies (Deutsche Interdisziplinäre Vereinigung für Intensiv- und Notfallmedizin [DIVI] and Deutsche Gesellschaft für Internistische Intensivmedizin und Notfallmedizin [DGIIN]) in March and April 2017. Participants were told to complete the survey only once per ICU, which was partly controlled by using cookies. No incentives were offered for completing the survey.

### Statistical analysis

Survey results were described by percentages for categorical variables and by median and interquartile range for continuous variables, since these were nonnormally distributed. To calculate the average rounding time per bed we used a bed occupancy of 80% and calculated the average rounding time per patient using the almost identical geometric mean for calculation, as the data was nonnormally distributed. Because of the exploratory and descriptive nature of the study no statistical tests were conducted. If participants did not answer single survey items, they were not excluded from the sample and their available data was used. All analyses were conducted using the statistical program JMP 14.2[8].

## Results

### Sample characteristics

In total, 390 participants completed the survey. In 2017 a total of 1160 hospitals with ICUs were registered in Germany [9]. The ICUs surveyed covered a broad range of medical and surgical specialties, often in a multidisciplinary setting (Table 1). In smaller hospitals, ICUs were often organized as mixed interdisciplinary ICUs, whereas in university hospitals, more specialized ICUs dominated. A fraction of ICUs specialized in fields such as neurology, neurosurgery, pediatrics or neonatology. Cardiology patients were the most common subgroup and treated in 65% of all ICUs.

**Table 1:** Specialties represented in the ICUs participating in the survey

Medical specialty	*n*	%
Cardiology	255	65
Gastroenterology	236	61
Pulmonology	234	60
General surgery	207	53
Trauma surgery	185	47
Nephrology	183	47
Oncology	143	37
Other surgical subjects	131	34
Neurology	131	34
Other internal medicine subjects	99	25
Cardiac surgery	84	22
Neurosurgery	81	21
Pediatric surgery	16	4
Reconstructive surgery	15	4
Pediatrics	15	4
Gynecology	11	3
Neonatology	9	2
Urology	4	1
ENT	4	1

Participants in the survey represented ICUs at university hospitals (25%), tertiary hospitals (23%), secondary hospitals (36%) and primary hospitals (16%).

The positions of the participants and their medical trainings are shown in Table 2. Participants with more than one specialty could check more than one answer, depending on their trainings.

**Table 2:** Information about the participant

Role of the participant	*n*	%
Head of the ICU	130	33
ICU attending	110	28
Head of department	91	23
Attending physician	25	6
ICU fellow	15	4
Intensivist	11	3
Other	6	2
Chief nurse	2	1
Medical training of the participant	*n*	%
Internal intensive-care medicine	167	43
Anesthesia	158	41
Cardiology	75	19
Pulmonology	32	8
Surgical intensive-care medicine	23	6
Neurology	19	5
Cardiac surgery	14	4
Gastroenterology	14	4
Pediatrics	15	4
Nephrology	11	3
Other internal medicine subjects	11	3
General surgery	6	2
Trauma surgery	6	2
Oncology	7	2
Neurosurgery	8	2
Neonatology	7	2
Interdisciplinary intensive-care medicine	6	2
Reconstructive surgery	2	1
Emergency medicine	4	1
Infectiology	3	1
Pediatric surgery	1	0
Other surgical subjects	1	0

The ICUs were reported to provide in median 12 intensive care beds (Q_1_: 9; Q_3_: 16). Intermediate care (IMC) beds were integrated in 57% (*n* = 223) of these ICUs, with a median of 6 beds (Q_1_: 4; Q_3_: 10, *Q* quartile).

### Rounding process

The following data represents the main daily ward round on ICUs. In most cases, this main daily ward round on the ICU was reported to take place during the morning shift (55%) or the handover from the night to the morning shift (35%). The most common starting time was between 7 and 8 a.m., with 91% of the participants stating that the ward round starts on time. The median duration of a round was 60 min (Q_1_: 40 min; Q_3_: 90 min). An average rounding time per patient of 4.3 min was calculated from the data.

In 149 (38%) of all ICUs, the respondents stated that their staff splits into teams for separate ward rounds. In most of these cases (71%) rounds were performed by two teams.

Almost all (96%) of the participants stated that rounding includes a bedside visit. The patient’s medical chart was reviewed in 84% immediately prior to or while visiting each patient, in 8% this occurred separately before and in 6% after seeing all patients. The main locations of this chart review are shown in Table 3. The chart review took place in 72% of ICUs in the patient’s room, in 33% in the area in front of the patient’s room and in 36% in other locations in the ward such as a doctor’s room (multiple answers possible).

**Table 3:** Location of the medical chart review

Location	*n*	%
Patient’s room	282	72
Area in front of the patient’s room	129	33
Doctor’s room	79	20
Ward center	50	13
Conference room	6	2
Ward lounge	3	1
Other	1	0

Regarding daily goals, participants stated that they are always (55%) or usually (39%) set during rounds. These goals were said to be documented for all (38%) or most (42%) of the patients.

**Fig. 1:**
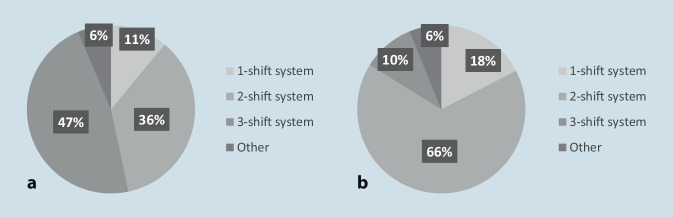
Resident’s shift systems on **a** weekdays and **b** weekends

In 57% of ICUs, a second daily ward round took place on the same day but was reported to differ from the first. In 29% of the ICUs, a second ward round was performed, in an identical process as the first round. Only 14% of respondents indicated holding only a single ward round per day. On weekdays, 47% of the ICU residents were reported to work in three-shift system with three handovers, while 36% worked in a two-shift system with two handovers. On weekends, a two-shift system with two handovers was most common (Fig. 1).

### Team structure during the round

To better describe the structure and members of each rounding team, participants were asked to state if individual members are always, usually, sometimes, rarely or never participating during rounds using Likert-scaled questions (Table 4). The data shows that residents, attending intensivists, ICU nurses and heads of the ICU were most common members of rounding teams. The results of the survey show that other medical professions such as respiratory therapists, physiotherapists, pharmacists and microbiologists never or only infrequently attended ICU rounds. Respiratory therapists are a relatively new profession in Germany, and 47% of all ICUs reported not to have one in their team. In contrast, while being an integral part of the ICU team, physiotherapists did in most ICUs not participate in daily rounds. Pharmacists did not attend rounds in 68% of those surveyed, and neither did 49% of microbiologists or other infectious disease specialists.

**Table 4:** Participants ICU rounds on weekdays

Member of ICU team	Not existing	Never	Rarely	Sometimes	Usually	Always
Resident	9 (2%)	0 (0%)	3 (1%)	6 (2%)	50 (13%)	322 (83%)
ICU attending	34 (9%)	4 (1%)	8 (2%)	28 (7%)	90 (24%)	217 (57%)
ICU nurse	0 (0%)	2 (1%)	14 (4%)	35 (9%)	141 (36%)	198 (51%)
Head of the ICU	57 (15%)	5 (1%)	12 (3%)	19 (5%)	107 (28%)	183 (48%)
ICU fellow	32 (8%)	5 (1%)	26 (7%)	131 (34%)	95 (24%)	100 (26%)
Attending physician	78 (21%)	33 (9%)	46 (13%)	72 (20%)	64 (18%)	70 (19%)
Head of department	2 (1%)	62 (16%)	82 (21%)	78 (20%)	95 (24%)	69 (18%)
Chief nurse	2 (1%)	33 (8%)	86 (22%)	113 (29%)	92 (24%)	64 (16%)
Physicians of other subjects	10 (3%)	35 (9%)	84 (22%)	115 (29%)	88 (23%)	58 (15%)
Intensivist	99 (26%)	17 (5%)	30 (8%)	94 (25%)	80 (21%)	56 (15%)
Medical student	36 (9%)	24 (6%)	98 (25%)	154 (39%)	60 (15%)	18 (5%)
Respiratory therapy	178 (46%)	65 (17%)	46 (12%)	55 (14%)	30 (8%)	16 (4%)
Physiotherapy	2 (1%)	152 (39%)	117 (30%)	84 (22%)	24 (6%)	11 (3%)
Microbiology, virology, etc.	42 (11%)	150 (38%)	72 (18%)	111 (28%)	12 (3%)	3 (1%)
Pharmacy	49 (13%)	216 (55%)	55 (14%)	63 (16%)	5 (1%)	2 (1%)
Ethics consulting	42 (11%)	187 (48%)	125 (32%)	32 (8%)	2 (1%)	2 (1%)

### Weekend rounds

On weekends, 70% of all ICUs reported one ward round per day with the attendance of an intensivist or an attending in the role of decision-maker, 9% reported two ward rounds per day and 18% only one ward round with the presence of a decision-maker over the whole weekend (in 77% on Sundays). In 3% of the ICUs, no ward round with a decision-maker was performed on weekends. Our data shows that higher qualified, and board-certified intensivists as decision-makers (heads of department, head of the ICU, ICU attending) were less available during weekends (e.g., 57% vs 28% for the invariable attendance of an ICU attending).

### Rounds of external teams

Concerning additional rounds with teams from other specialties, the specialty most frequently reported to be rounding separately on the ICU was general surgery (49%). The most frequently mentioned ICU team members joining the rounds of external staff were the attending intensivist (56%) and ICU residents (59%). The median time spend for these external rounds by the ICU team was 30 min (Q_1_: 15 min; Q_3_: 45 min).

### Documentation

**Fig. 2:**
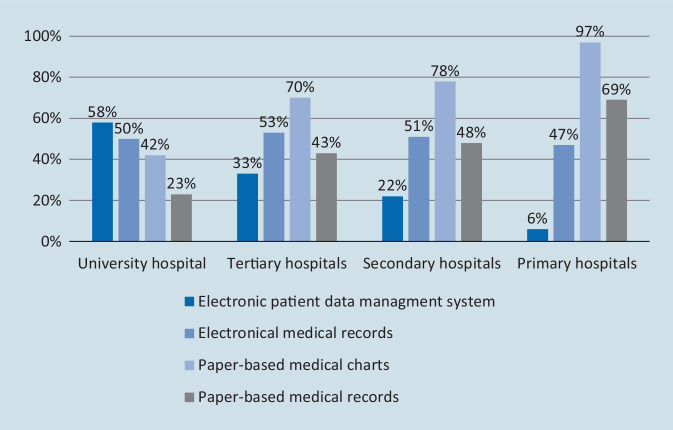
Types of medical documentation systems used during rounds related to hospital size. Multiple answers were possible

Most participating ICUs used paper-based medical charts (70%) for documentation purposes, whereas electronic medical charts (patient data management systems, PDMS) were used in only 31% of ICUs (Table 5). However, there was a wide variation depending on the hospital size (Fig. 2). The smaller the hospital, the more the charts were paper based. In contrast, electronic medical records (providing access to various medical documents) were used in 51% and paper-based medical records in 44% of the rounds. The general availability and use of technological information sources were also surveyed (Table 5). Laboratory information systems, radiology information systems and microbiology information systems were used in the majority of rounds.

**Table 5:** Use of electronic information systems during rounds

Use of electronical devices	Not existing	Never	Rarely	Sometimes	Usually	Always
LIS (laboratory information system)	6 (2%)	6 (2%)	14 (4%)	20 (5%)	61 (16%)	281 (72%)
RIS (radiology information system)	4 (1%)	3 (1%)	16 (4%)	29 (7%)	93 (24%)	245 (63%)
Microbiology information system	25 (7%)	9 (2%)	19 (5%)	39 (10%)	97 (25%)	195 (51%)
HIS (hospital information system)	46 (12%)	14 (4%)	39 (10%)	43 (11%)	74 (19%)	166 (43%)
PDMS (patient data management systems)	218 (60%)	12 (3%)	6 (2%)	10 (3%)	12 (3%)	106 (29%)
Drug information system	11 (3%)	27 (7%)	89 (24%)	106 (28%)	56 (15%)	88 (23%)
PubMed	48 (13%)	78 (21%)	102 (27%)	91 (24%)	13 (3%)	46 (12%)
Electronical medical books	69 (18%)	63 (17%)	94 (25%)	99 (26%)	17 (4%)	37 (10%)
Electronical medical journals	51 (14%)	75 (20%)	101 (27%)	92 (25%)	21 (6%)	35 (9%)
Other internet-based programs	42 (11%)	55 (15%)	103 (28%)	107 (29%)	32 (9%)	32 (9%)
Smartphone Apps	60 (16%)	42 (11%)	86 (23%)	118 (32%)	33 (9%)	28 (8%)

## Discussion

We performed an online survey to gather data about the practice of rounding in German ICUs over four levels of care. Our survey shows that on most German ICUs, ward rounds take place in a very traditional manner; at the bedside with physicians and nurses present using predominantly paper-based medical charts. Other professions or medical specialists such as physiotherapists, respiratory therapists, pharmacists or microbiologists were not reported to be a regular part of the daily rounding team on most ICUs.

There was considerable variation of rounding practices across ICUs regarding time schedule, participating members of the rounding team, location of chart review, weekend rounds and medical documentation used, especially in terms of computers and medical software.

From our data we calculated an average rounding time of 4.3 min per patient. This includes checking the medical chart, making further decisions about the treatment strategy and in most cases, visiting patients in their rooms and setting daily goals. The Canadian survey of ICU rounds by Holodinsky et al. reported a median round duration of 168 min, with a median of 15 min per patient [5]. A US study compared two concepts of structured interdisciplinary bedside rounds and reported total rounding times per patient of 16.9 min and 22.4 min [10]. Thus, it appears that ICU rounds in Germany compared to ICU rounds in Anglo-American countries are shorter, not as detailed and less interdisciplinary. On the contrary, overly long rounds which occupy the ICU team for an extended period of time can potentially lead to rounding fatigue and can cause a delay of other vital tasks in the ICU such as admissions, discharges, transports and procedures.

Our survey provides no data on the possible impacts of the shorter duration of German ICU rounds such as on the quality of patient care, or communication and teaching within the ICU team. In the Canadian survey, the majority of participants reported having efficient (79%) and equitable rounds (88%). The latter means that patients received the time and attention that physicians thought was required. Only a minority of the participants complained about spending too much time on minor issues [5]. There is also evidence that daily multidisciplinary ICU rounds are associated with improved patient safety and lower patient mortality [11, 12]. The presence of a pharmacist during ICU rounds is widespread in Anglo-American countries and can reduce drug-related events and save costs [13–15]. In Canada, pharmacists attended ICU rounds regularly in 85% and respiratory therapists in 89% of those surveyed [5]. The German Interdisciplinary Society of Intensive Care Medicine (DIVI) has defined daily multiprofessional and interdisciplinary clinical rounds with documentation of daily goals as the number 1 quality indicator for ICUs [1]. In reality, however, this seems to be reduced to the presence of physicians and nurses. Furthermore, daily goals are documented inconsistently.

In Germany, 29% of respondents stated that on weekdays their ICU performs a second ward round instead of having one long morning round, mostly in the afternoon, which is identical to the morning round. In 57% of the ICUs, the format of the second daily ward round differed from the first. Only 14% of the ICUs reported a single ward round per day. In addition, members of the ICU team spent an average of 30 min rounding with other external teams such as general surgeons. Therefore, daily time required for extra rounds and handovers must be considered in addition to the main morning round, however, this survey did not allow for the summation of time taken for rounds and handovers on weekdays and weekends.

A 3-shift system with three rounds or handovers was applied by 47% of the ICUs on weekdays, but only 10% on weekends. On weekends a 2-shift system dominates with 66% vs 36% on weekdays. Corresponding to this, most ICUs performed only one daily round on weekends.

Due to the limited amount of scientific data and the variability of the rounding practice, the optimal ICU round in terms of quality of care, patient safety, overall ICU performance, efficiency, teaching quality and team satisfaction still must be defined. It should be investigated whether it is possible to achieve all these, sometimes conflicting goals within a time frame of approximately 8–10 min per patient or a total duration of 2 h.

This process could be supported by an electronic patient data management system (PDMS) that is specifically designed to provide optimal functionality for rounds. The advantages of PDMS in ICUs have been emphasized in the literature [16]. The usability of PDMS in terms of support for medical decision making during rounds or ICU admissions, however, still is far from optimal [12, 17–19]. A PDMS should allow a rapid, systematic and complete visual check of all relevant patient data including labs, imaging, ECGs, etc. paired with appropriate alerts or highlighting of pathology values and automatic drug safety features, maybe in the future also with the added support of machine learning technology and artificial intelligence [20, 21]. The verbal discussion can then focus on the relevant individual patient problems. Multiple computers allow for the multitasking of team members with simultaneous order entry, documentation of daily goals or an information search on the Internet. An optimized electronic chart review using multiple large-screen computers is challenging to perform at the bedside or in the hallway and requires a specially dedicated and equipped area in the ICU. An alternative approach would be to perform a complete electronic chart review in such an area before seeing the patients at the bedside. This has the potential to improve the quality and efficiency of the chart review but makes it more difficult to integrate the bedside nurse. This approach, however, has not yet been systematically compared to a traditional bedside round in the ICU setting.

Only 31% of the ICUs in our survey used a PDMS for chart review during rounds. The general availability of a PDMS varied with the size of the hospital and was at its highest in university hospitals (58%) and lowest in small hospitals (6%). We found no representative data about the availability of PDMS in ICUs of other developed countries. A recent German international benchmarking study, however, found Germany lagging far behind in terms of digital health implementation [22]. Investments in digital health technology are largely underfinanced in the German health system, where reimbursement is mainly based on case fees based on diagnosis-related groups (DRG). Intensivists in Germany have suggested a change to the hospital financing system, which also includes development and investment programs for health information technology (IT) systems, including PDMS [23, 24].

Another issue highlighted by our survey relating to the reimbursement system for intensive care medicine in Germany is the presence of a board-certified intensivist as a decision-maker/supervisor on rounds 7 days per week. The operation and procedure key (OPS) 8‑98f [25] was introduced after our survey in 2018 and is required for reimbursement for more costly and complex intensive care treatments. It was initially designed as a criterion for university and other tertiary care hospitals. Medium-sized hospitals also trying to bill treatments using OPS must prove the presence of an intensivist during all daily rounds. It therefore follows that these numbers have probably increased since our survey. In our survey, 21% of the participants stated that only one or even no ward rounds with the attendance of a decision-maker took place during weekends. Furthermore, the results showed that the attendance of highly qualified decision-makers like the ICU attending decreases during weekends.

This survey has several limitations. Participation was voluntary, and we achieved the participation of 33% of hospitals with ICUs in Germany, if every participant is representing exactly one ICU. The number of total ICUs, however, is higher because large hospitals often have several specialized ICUs. Rounding processes were only indirectly measured based on the generalized experiences of the survey participants. In addition, only data regarding the structure and the processes of rounds was gathered. A questionnaire or interview, including the subjective perception of different qualitative aspects of rounds addressing different participants of the rounding team, was beyond the scope of this survey. We also had no data to correlate rounding practices with quality of care and patient outcome as well as to analyze how disease severity and patient complexity influenced rounding practice and time spent per patient.

## Conclusion

Our survey provides the first collection of a wide variety of data regarding rounding structures and processes across German ICUs. Most ICUs carry out a traditional bedside round which is shorter and less interdisciplinary than those in other countries. We hope that our data will stimulate further projects and studies to develop and investigate optimal rounding concepts while including the aspects of interdisciplinary teamwork, integration of modern health information technology, time efficiency and overall ICU performance.

## Declarations

All procedures performed in studies involving human participants or on human tissue were in accordance with the ethical standards of the institutional and/or national research committee and with the 1975 Helsinki declaration and its later amendments or comparable ethical standards. Informed consent was obtained from all individual participants included in the study. The study was reviewed by the ethics committee of the University of Tübingen, which waived the need for informed consent.
